# Supplementary material for: The Thiol Group Reactivity and the Antioxidant Property of Human Serum Albumin Are Controlled by the Joint Action of Fatty Acids and Glucose Binding
Source: Int J Mol Sci. 2024 Feb 16;25(4):2335. doi: 10.3390/ijms25042335 (PMC10889162; doi:10.3390/ijms25042335)
Supplement: Supplementary file 1 [file ijms-25-02335-s001.zip › ijms-2853400-Supplementary data.pdf]

Albumin thiol group content and reactivity are under control of joint action of fatty acids and glucose binding

Tamara Uzelac[1§], Katarina Smiljanić[1], Marija Takić[2], , Ivana Šarac[2], Gordana Oggiano[2], Milan Nikolić[1] and Vesna Jovanović[1§*]

[1]*University of Belgrade - Faculty of Chemistry, Department of Biochemistry and Centre of Excellence for Molecular Food Sciences, Studentski trg 12-16, 11158 Belgrade, Serbia*

[2]*University of Belgrade, Institute for Medical Research, Centre of Research Excellence in Nutrition and Metabolism, National Institute of Republic of Serbia, Tadeuša Košćuškog 1, 11000 Belgrade, Serbia*

[§]These authors contributed equally to this work.

* Corresponding author:
Vesna Jovanović, PhD
University of Belgrade – Faculty of Chemistry
Department of Biochemistry & Center of Excellence for Molecular Food Sciences
Studentski trg 12-16, Belgrade 11158, Serbia
E-mail: vjovanovic@chem.bg.ac.rs; Tel/Fax: +381 11 3336 676

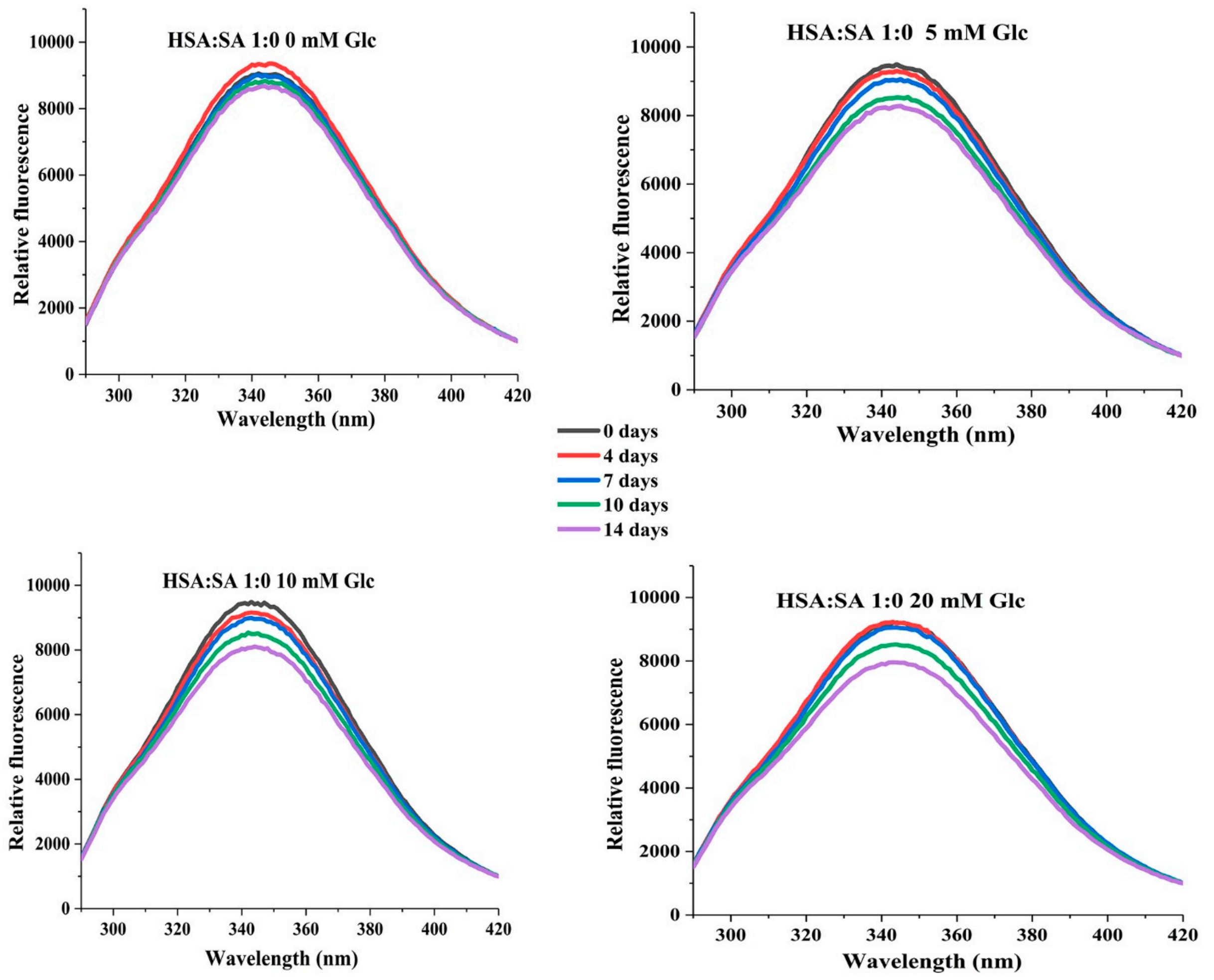

Figure S1. The effects of glucose concentrations (5, 10 and 20 mM) on the normalized Trp214 fluorescence intensity during 14 days of incubation of HSA in the absence of stearic acid (SA), HSA:SA 1:0. Before collecting spectra, HSA samples were diluted to the final concentration of 2 μM. Spectra were recorded at 37°C after excitation at 280 nm.

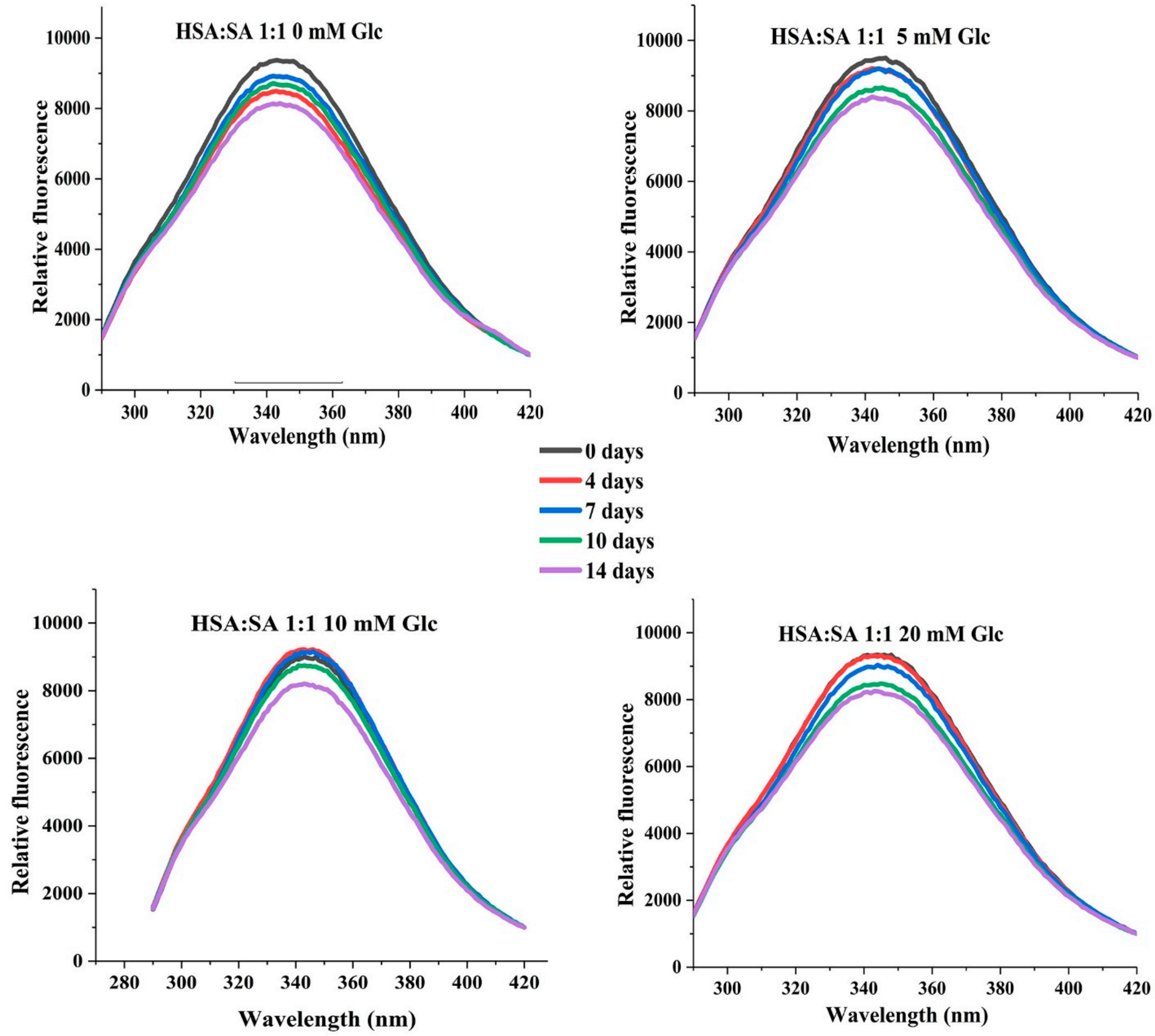

Figure S2. The effects of glucose concentrations (5, 10 and 20 mM) on the normalized Trp214 fluorescence intensity during 14 days of incubation of HSA in the presence of stearic acid (SA), HSA:SA 1:1. Before collecting spectra, HSA samples were diluted to the final concentration of 2 μM. Spectra were recorded at 37°C after excitation at 280 nm.

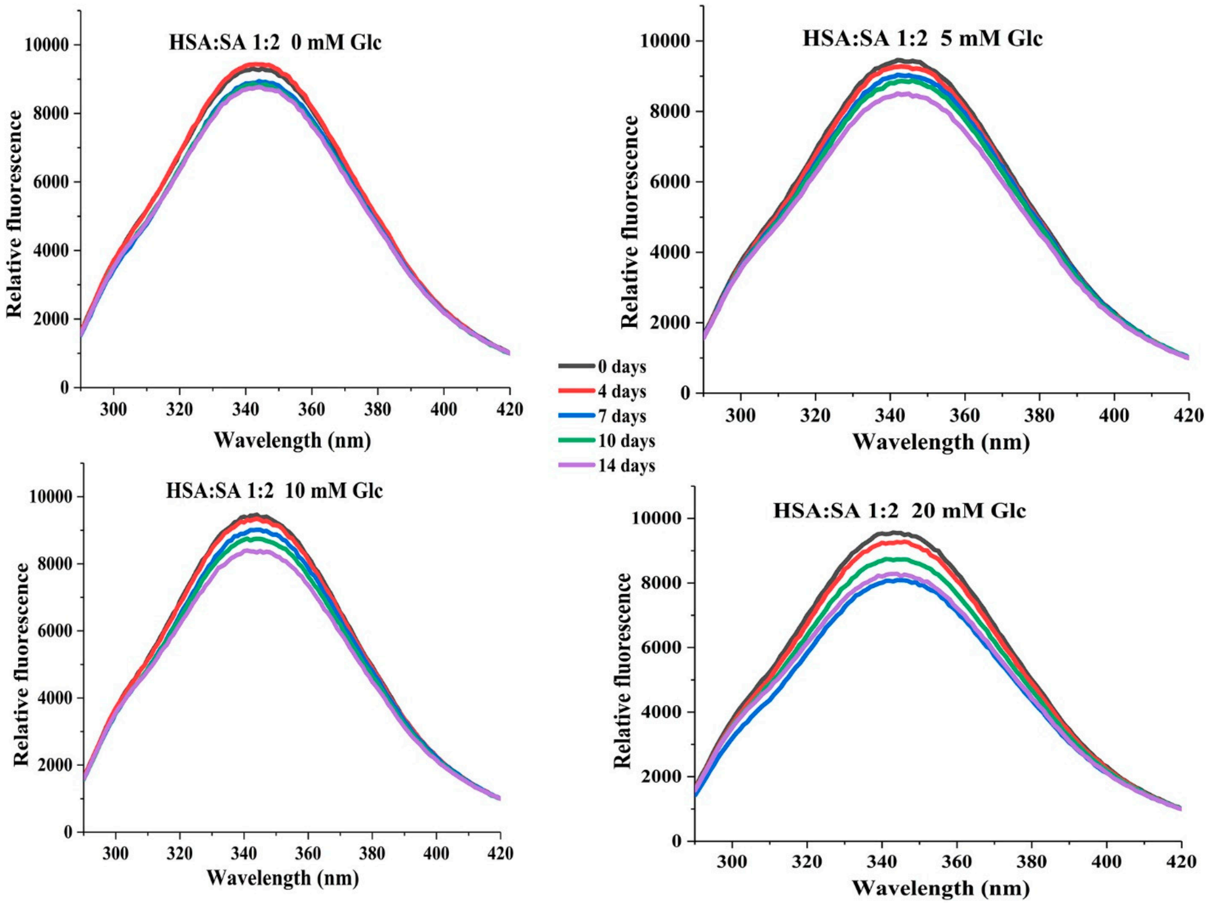

Figure S3. The effects of glucose concentrations (5, 10 and 20 mM) on the normalized Trp214 fluorescence intensity during 14 days of incubation of HSA in the presence of stearic acid (SA), HSA:SA 1:2. Before collecting spectra, HSA samples were diluted to the final concentration of 2 μM. Spectra were recorded at 37°C after excitation at 280 nm.

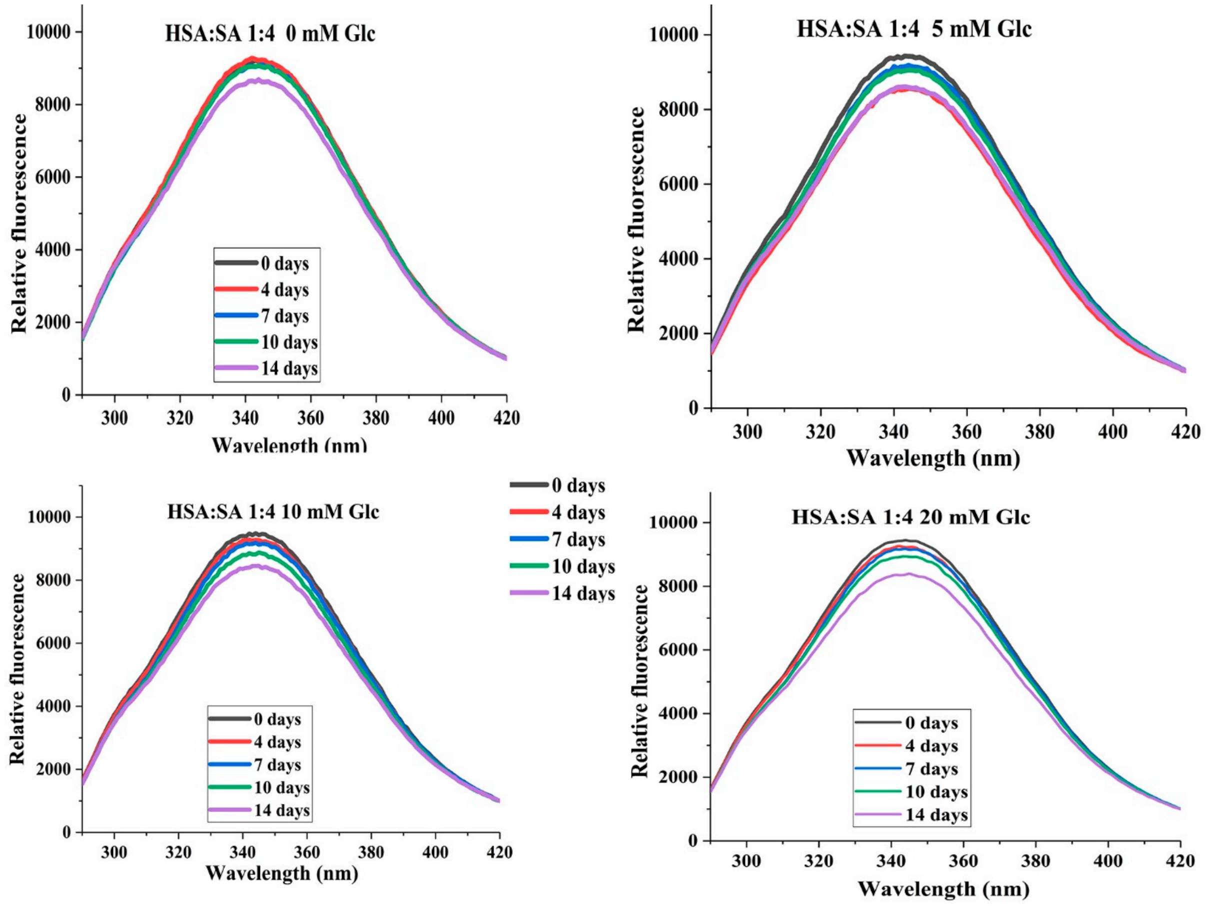

Figure S4. The effects of glucose concentrations (5, 10 and 20 mM) on the normalized Trp214 fluorescence intensity during 14 days of incubation of HSA in the presence of stearic acid (SA), HSA:SA 1:4. Before collecting spectra, HSA samples were diluted to the final concentration of 2 μM. Spectra were recorded at 37°C after excitation at 280 nm.

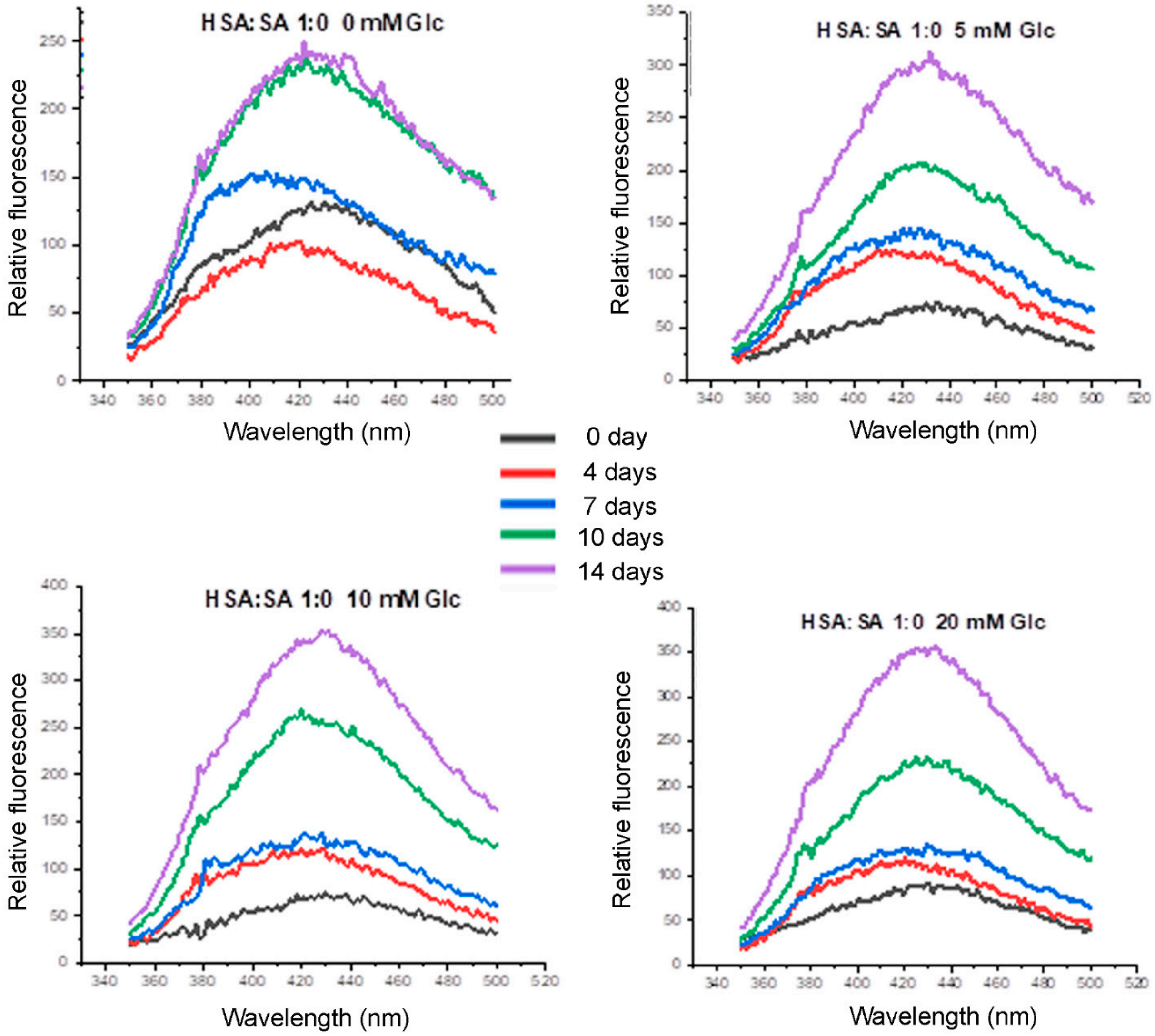

Figure S5. The effects of glucose concentrations (5, 10 and 20 mM) on the formation of AGEs during 14 days of incubation of HSA in the absence of stearic acid (SA), HSA:SA 1:0. Before collecting spectra, HSA samples were diluted to the final concentration of 2 μM. Spectra were recorded at 37°C after excitation at 335 nm.

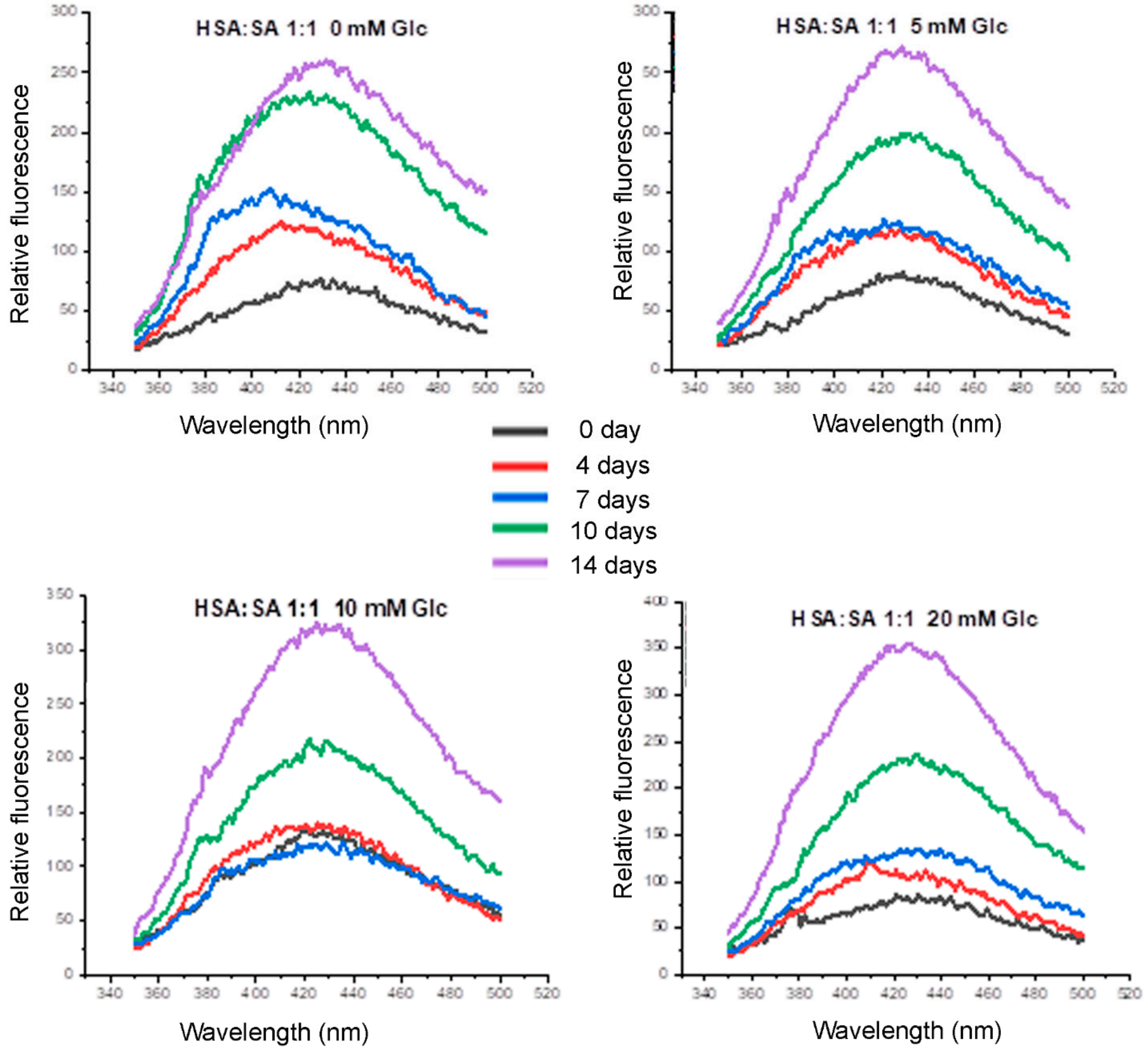

Figure S6. The effects of glucose concentrations (5, 10 and 20 mM) on the formation of AGEs during 14 days of incubation of HSA in the presence of stearic acid (SA), HSA:SA 1:1. Before collecting spectra, HSA samples were diluted to the final concentration of 2 μM. Spectra were recorded at 37°C after excitation at 335 nm.

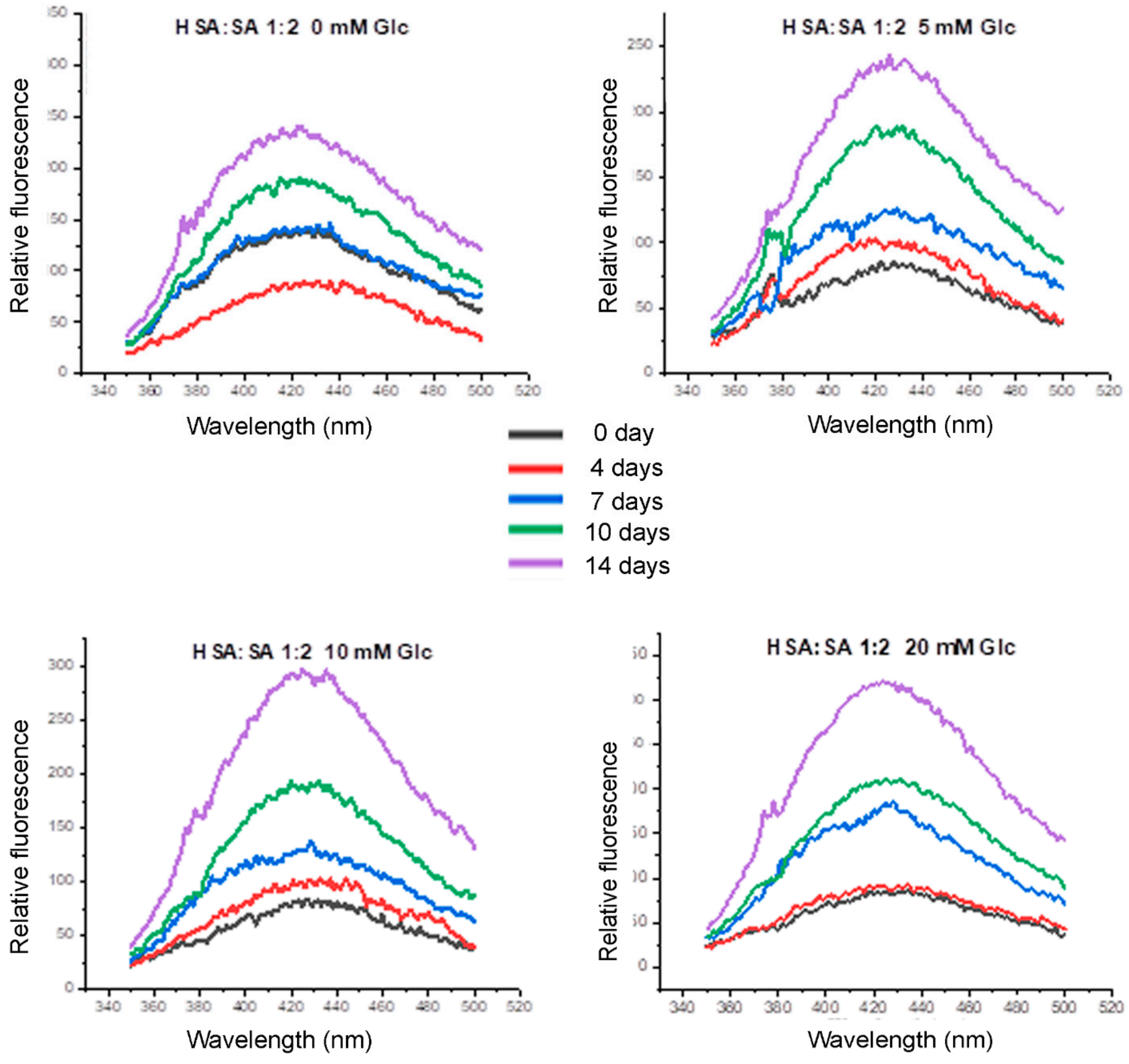

Figure S7. The effects of glucose concentrations (5, 10 and 20 mM) on the formation of AGEs during 14 days of incubation of HSA in the presence of stearic acid (SA), HSA:SA 1:2. Before collecting spectra, HSA samples were diluted to the final concentration of 2 μM. Spectra were recorded at 37°C after excitation at 335 nm.

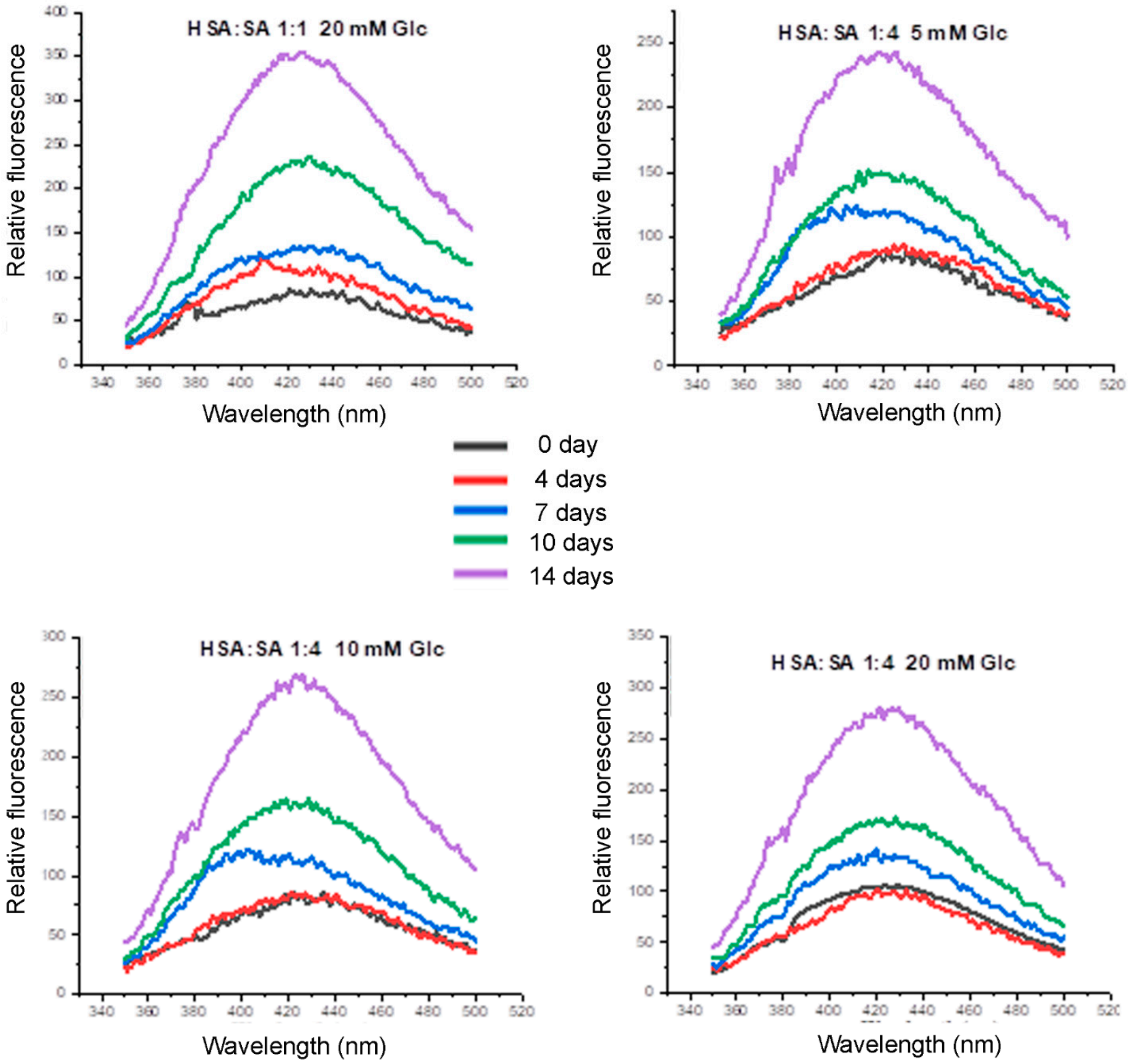

Figure S8. The effects of glucose concentrations (5, 10 and 20 mM) on the formation of AGEs during 14 days of incubation of HSA in the presence of stearic acid (SA), HSA:SA 1:4. Before collecting spectra, HSA samples were diluted to the final concentration of 2 µM. Spectra were recorded at 37°C after excitation at 335 nm.
